# Longitudinal characterization of olfactomedin-4 expressing neutrophils in pediatric patients undergoing bone marrow transplantation

**DOI:** 10.1371/journal.pone.0233738

**Published:** 2020-05-29

**Authors:** Julie E. Stark, Amy M. Opoka, Lin Fei, Huaiyu Zang, Stella M. Davies, Hector R. Wong, Matthew N. Alder

**Affiliations:** 1 Division of Critical Care Medicine, Department of Pediatrics, University of Cincinnati College of Medicine, Cincinnati Children's Hospital Medical Center, Cincinnati, OH, United States of America; 2 University of Cincinnati Department of Biostatistics and Epidemiology, Cincinnati, OH, United States of America; 3 Division of Bone Marrow Transplant and Immune Deficiency, Cancer and Blood Diseases Institute, Cincinnati Children’s Hospital Medical Center, Cincinnati, OH, United States of America; Charles P. Darby Children's Research Institute, UNITED STATES

## Abstract

Sepsis is an important cause of morbidity and mortality in pediatric patients. Increased expression of olfactomedin-4 (OLFM4), a glycoprotein contained within a subpopulation of neutrophils, has been associated with complicated course in sepsis. The factors that regulate OLFM4 expression are unknown. Here, we followed children undergoing bone marrow transplantation (BMT) to document the percentage of neutrophils that express OLFM4 over time. This population was selected because of the ability to observe nascent neutrophils following engraftment, perform frequent blood sampling, and the children are at high risk for clinical complications that may associate with changes in percentage of OLFM4+ neutrophils. We found a surprising degree of variability of OLFM4 expression between patients. In the weeks following initial neutrophil recovery we also saw great variability in OLFM4 expression within individual patients, indicating that multiple external factors may modify OLFM4 expression. We identified decreased expression of CD64 (a marker associated with response to infection), in OLFM4+ neutrophils. This is the first study to demonstrate fluctuation in OLFM4 expression within patients and provides insight into possible mechanisms for OLFM4 regulation in nascent neutrophils.

## Introduction

Neutrophils are the most abundant white blood cell in peripheral blood and are the primary mobile arm of the innate immune system. Initially thought of as primarily phagocytes, there is now more appreciation for the roles of neutrophils in adaptive immune responses and healing [1, 2]. Patients who are neutropenic due to primary disease or therapy have increased susceptibility to infections, especially bacterial. Bone marrow transplantation (BMT) is a life-saving procedure for many children with genetic and hematologic diseases, but complete ablation of host marrow and replacement with HLA matched donor stem cells requires intensive chemotherapy that eliminates host hematopoietic cells and may result in organ injury. After BMT nascent donor-derived neutrophils are detectable around two weeks after transplantation. Lymphocyte recovery occurs weeks later, and may be incomplete for months, particularly if acute graft versus host disease (GVHD) occurs. Patients remain at significant risk of serious infection, including sepsis, for months after BMT.

Pediatric sepsis is a leading cause of death in children worldwide. Large epidemiologic studies in developed countries demonstrate that though sepsis is not the most common admitting diagnosis in these pediatric intensive care units (ICUs), it is disproportionately responsible for mortality and morbidity following admission [3]. Additional risk factors such as cancer or BMT are independent risk factors for mortality in pediatric sepsis [4]. Inherent heterogeneity within patients who present with diverse pathophysiologies make treating sepsis difficult. Patients with seemingly similar infections may have dramatically different outcomes. Heterogeneity arises from many causes, one of which is heterogeneity within immune cells, including neutrophils.

Neutrophil heterogeneity has been largely attributed to differential activation states of a single neutrophil population, such as resting, activated, or aged neutrophils [5, 6]. Remarkably, olfactomedin 4 (OLFM4) demonstrates a binary expression pattern within neutrophils either expressing high amounts of OLFM4 or none during both health and active infection. OLFM4 is expressed intracellularly, within the specific granules of neutrophils [7, 8]. It is unclear if OLFM4 is a single gene whose expression differs between OLFM4+ and OLFM4- neutrophils or if OLFM4 expression marks a unique subpopulation of neutrophils with an inherently different transcriptional signature and functionality. In humans, about 25% of neutrophils are OLFM4+, but there is a range, with some patients expressing OLFM4 in as few as 5%, and others in over 50% of neutrophils. Previous investigators have noted that individual healthy adults maintain a stable percentage of OLFM4+ neutrophils over time, but little else is known about the natural course and regulation of OLFM4+ neutrophils [8]. Increased transcription of OLFM4 from whole blood samples is associated with increased disease severity in adults with acute respiratory distress syndrome and children with respiratory syncytial virus [9, 10]. Our interest in OLFM4 arose when transcriptome analysis demonstrated that OLFM4 was one of the most upregulated genes in pediatric sepsis, and the most upregulated gene in pediatric sepsis with complicated course (defined as death at 28 days or failure of two or more organ systems at 7 days) [11, 12]. A high percentage of OLFM4+ neutrophils at the time of admission to the intensive care unit also predicts poor outcome in pediatric sepsis patients [13]. However, it is unclear if these patients always expressed high levels of OLFM4 at baseline, or if sepsis or organ injury induced increased expression of OLFM4.

The objectives of this study were two-fold: first, to assess longitudinal changes in the percentage of nascent neutrophils that are OLFM4+ after BMT and second, to test if changes in percentage of OLFM4+ neutrophils signaled important clinical change. BMT recipients were selected to allow for the ability to assess the first waves of nascent neutrophils during engraftment. Given these patients undergo frequent blood sampling, we were able to follow over time and assess for changes in OLFM4 expression. Finally, given the immune suppression and increased susceptibility to serious infections, as well as other morbidities like GVHD and thrombotic microangiopathy (TMA), we sought to correlate these clinical events with changes in OLFM4 expression.

## Methods

### Patient sample selection

This study was approved by the Institutional Review Board at Cincinnati Children’s Hospital. Written consent was obtained from patients and families through the Cancer and Blood Diseases Institute at Cincinnati Children’s Hospital, which allowed use of residual samples for further research. 50 consecutive patients who consented to the protocol from December 2016 through May 2018 were included regardless of diagnosis (malignant versus non-malignant) or transplant type (autologous versus allogeneic). Complete blood cell counts (CBCs) were collected at least a week prior to BMT, at the time of BMT, and weekly for the following 5 weeks. A final sample was collected at 100 days post-transplant when available. Healthy control samples were obtained from siblings of patients who presented to the hospital for a clinical appointment who consented for donation of a small amount of blood for research.

### Flow cytometry

Flow cytometry was performed on blood samples using standard flow cytometric protocols. Samples underwent centrifugation, and plasma was removed and stored at -80°C. Based on white blood cell count, samples were aliquoted to approximately 1 million white blood cells per tube. Samples underwent red blood cell lysis, and cells were washed with FACS buffer (1xPBS with 0.1% BSA and 1mM sodium azide). Fc receptors were blocked with 10% human serum in FACS buffer. Cells were incubated with the following surface antibodies: CD177 (Life Technologies, Frederick, Maryland), CD66b (BD Biosciences, San Jose, California), CD3 (Invitrogen, San Diego, California, USA), CD64 (BD Biosciences, San Jose, California, USA), CD16 (BD Biosciences, San Jose, California, USA), CD11b (Tonbo Biosciences, San Diego, California, USA), and CD62 ligand (CD62L; Thermo Fischer Scientific, San Diego, California, USA). Following incubation, cells were washed with FACS and fixed with 2% PFA. They were then permeabilized with ICCS buffer (1xPBS with 10mM HEPS, 0.1% BSA, 0.1% saponin). A second block was performed with 10% human serum in ICCS buffer. Intracellular staining was performed with rabbit anti-OLFM4 antibody (Santa Cruz Biotechnology, Santa Cruz, California) followed by anti-rabbit secondary conjugated to PE (Jackson ImmunoResearch, West Grove, Pennsylvania). All samples were washed with ICCS and FACS buffers and run on the same BD FACSCanto II analyzer. White blood cells were gated using forwards scatter and side scatter, and neutrophils were identified using CD66b. Percentage of cells expressing OLFM4 was recorded, along with mean fluorescence intensity (MFI) of cell surface markers. To ensure the anti OLFM4 antibody was indeed staining OLFM4, we compared it with three other antibodies specific for OLFM4 and found that all four antibodies identified the same population of neutrophils (S1 Fig).

### Serum OLFM4 concentration

Plasma samples were collected from each of the samples and stored at -80°C. Plasma OLFM4 concentration was then determined using a custom kit designed and validated by Millipore (MilliporeSigma Burlington, MA). Plasma was diluted 1:50 for these assays. Samples were evaluated on a Luminex200 analyzer.

### Patient information

Clinical data indicating organ injury were compiled for each patient including: creatinine, total bilirubin, alanine aminotransferase (ALT), aspartate aminotransferase (AST), and need for oxygen or mechanical ventilation. Where applicable, the date of onset of acute GVHD and TMA were determined by consensus by physicians within the Cancer and Blood Diseases Institute. White blood cell count and absolute neutrophil count (ANC) were recorded on the date that each CBC was obtained. Finally, the date and species of positive blood cultures were recorded.

### Statistical analysis

Statistical analysis was carried out using SigmaStat Software (Systat Software, San Jose, CA). Data are reported as percentages with aggerate descriptions using medians with interquartile ranges. For comparison between groups we used Mann-Whitney U test or Kruskal-Wallis one way ANOVA for weekly data. For correlation tests, we used Pearson Product Moment Correlation. Flow cytometric MFI group data were compared using Mann-Whitney U test. Logistic regression modelling was conducted using SAS 9.4 GLIMMIX. Because no prior comparisons in change in OLFM4 expression have been carried out, we made three different comparisons: The fold change in absolute OLFM4+ neutrophil count or OLFM4+ percentage were the independent variables and was assessed as change in 1) OLFM4+ neutrophils from baseline (first measurement following engraftment, typically two weeks following stem cell infusion) to the week of clinical change, 2) the week of clinical change compared to the prior and 3) the week of clinical change to the following week. The fold change in absolute OLFM4+ neutrophil counts were skewed due to initial low values so log10 of fold change was used for calculations. The binary dependent clinical outcomes were need for supplemental oxygen, need for mechanical ventilation, positive blood culture, onset of GVHD, and onset of TMA. AKI was defined using KDIGO (Kidney Disease Improving Global Outcomes) criteria and made binary with no kidney injury and KDIGO stage 1 counting as no kidney injury and stages 2 and 3 counting as acute kidney injury [14]. Bilirubin levels were grouped into scoring categories 2–4 used for P-SOFA (Pediatric Sequential Organ Failure Assessment) scoring, using bilirubin less than 1.2 as no injury and 2–4 as liver injury [15]. Since there were repeat observations nested within subjects, generalized linear mixed models of clinical outcomes were used to account for the correlation of data to percent OLFM4 change. Mixed-effects logistic regression was implemented for all binary outcomes. For logistic regression, we modeled the probability of not being in normal range for all clinical outcomes. The models were initially adjusted for gender and age and were then trimmed by removing non-significant adjusted covariates. Raw data used to derive graphs in this manuscript can be found in the supplementary data file.

## Results

**Fig 1:**
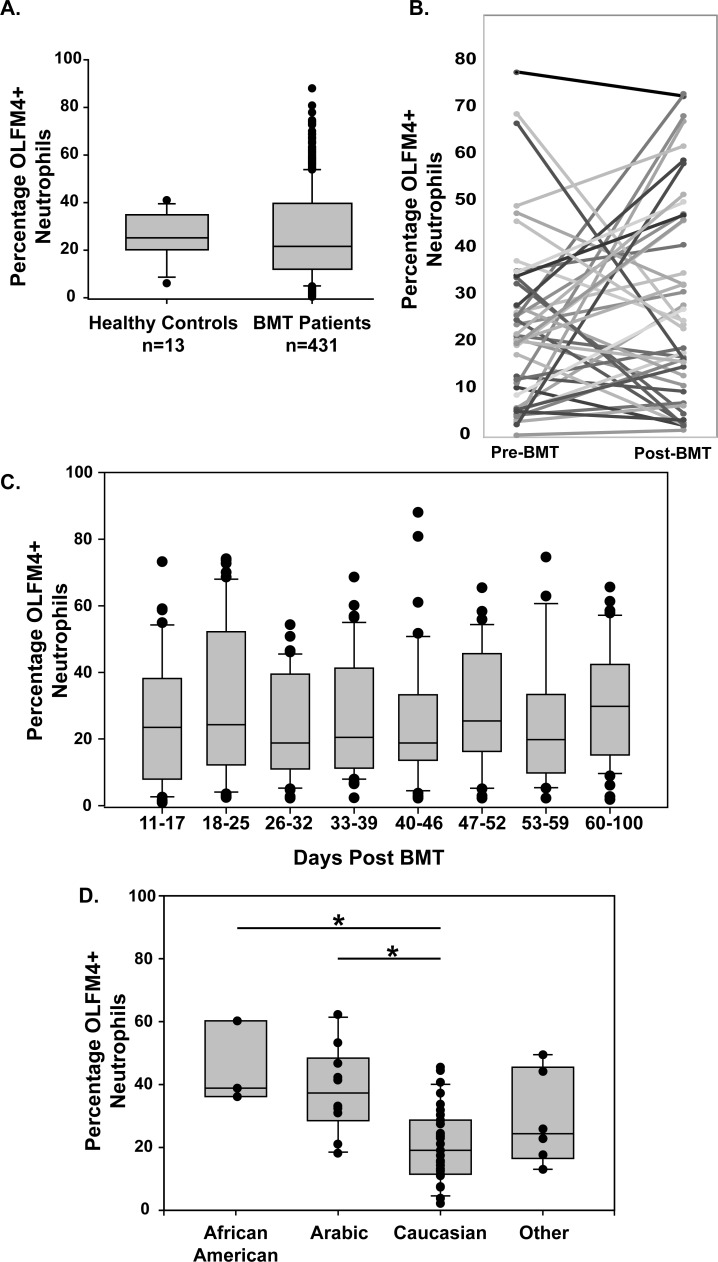
Percentage of OLFM4+ neutrophils in patients undergoing bone marrow transplantation. **A.** Box and whisker plots showing median, 25^th^ to 75^th^ percentile for each sample from healthy controls and from patients undergoing BMT. **B.** Last measured percentage of OLFM4+ neutrophils before myeloablative regiment and first measurement following engraftment. **C.** Box and whisker plots for percentage of OLFM4+ neutrophils each week following engraftment. **D.** Box and whisker plot showing average OLFM4 expression for all time points following engraftment for each patient grouped by race. One way analysis of variance of the groups showed statistically significant differences (denoted by *) between Arabic and Caucasian (p = 0.002) and African American and Caucasian (p = 0.01).

Consecutive pediatric patients scheduled for BMT, regardless of diagnosis, were approached for consent. Data from 50 patients are reported here. Patient demographics are described in Table 1. Weekly blood samples were collected and analyzed for OLFM4 expression within neutrophils starting two weeks prior to BMT and were followed to up to 100 days post BMT. A total of 473 samples from the 50 patients were collected, with data excluded on samples with white blood cell count less than 0.5x10^6^/mL, typically occurring between days 0 to 14 after stem cell infusion due to the conditioning regimen given prior to stem cell infusion. When assessing all samples together, the patients in this study demonstrated a wide range in percentage of OLFM4+ neutrophils, with a range of 0.4% to 88% positive. The median expression of all samples was 21.6%, which was not statistically different than healthy controls (25.2%; p = 0.53) (Fig 1A). To evaluate if host factors were responsible for determining percentage of OLFM4+ neutrophils, we assessed for correlation between percentage of OLFM4+ neutrophils before and after BMT for each patient using their last percentage prior to myeloablative therapy and their first measurement after 14 days and found no correlation (p = 0.11; Fig 1B). To test if there was a general trend of increase or decrease in percentage of OLFM4+ neutrophils following engraftment over time we evaluated all samples by week post-BMT and no significant difference was seen in percentage of OLFM4+ neutrophils on any given week (Fig 1C). We also measured the average percentage of OLFM4+ neutrophils from each patient using all data points following engraftment. When grouped by race, African American and Arabic patients had a higher percentage of OLFM4+ neutrophils compared to Caucasian patients (Fig 1D). Throughout the manuscript, we refer to the baseline measurement of percentage of OLFM4 positive neutrophils as the first measurement with WBC greater than 0.5x10^6^/mL following BMT. We tested for differences in average percentage of baseline OLFM4+ following transplantation between allograft and autograft transplant recipients and found no difference (29 +/- 21% vs 22 +/- 18%, respectively; p = 0.33). There was no correlation between baseline OLFM4+ percentage and age (R = -0.183, p = 0.21).

**Table 1:** Demographics of patients enrolled in the study.

		Number (percentage)
Gender		
	Male	32 (64)
	Female	18 (36)
Race		
	Caucasian	31 (62)
	Arabic	10 (20)
	African American	3 (6)
	Other	6 (12)
Age		
	0–4	18 (36)
	5–9	13 (26)
	10–14	6 (12)
	15–19	6 (12)
	≥20	7 (14)
Diagnosis		
	Non-malignant	31 (62)
	Malignant	19 (38)
Stem Cell Source		
	Autologous	11 (22)
	Matched related	9 (18)
	Matched unrelated	18 (36)
	Mismatched unrelated	11 (22)
	Haploidentical	1 (2)

Original diagnoses included: Fanconi anemia, Ewing sarcoma, aplastic anemia, acute myeloid leukemia, severe combined immunodeficiency, acute lymphocytic leukemia, Diamond Blackfan anemia, lymphoma, Wiksott Aldrich, sickle cell disease, CD40 ligand deficiency, neuroblastoma, beta thalassemia, germ cell tumor, Hurler’s syndrome, hemophagocytic lymphohistiocytosis, malignant metastatic rhabdoid tumor, Schwachman-Diamond syndrome, Paroxysmal nocturnal hemoglobinuria, and suprasellar high-grade glioma

**Fig 2:**
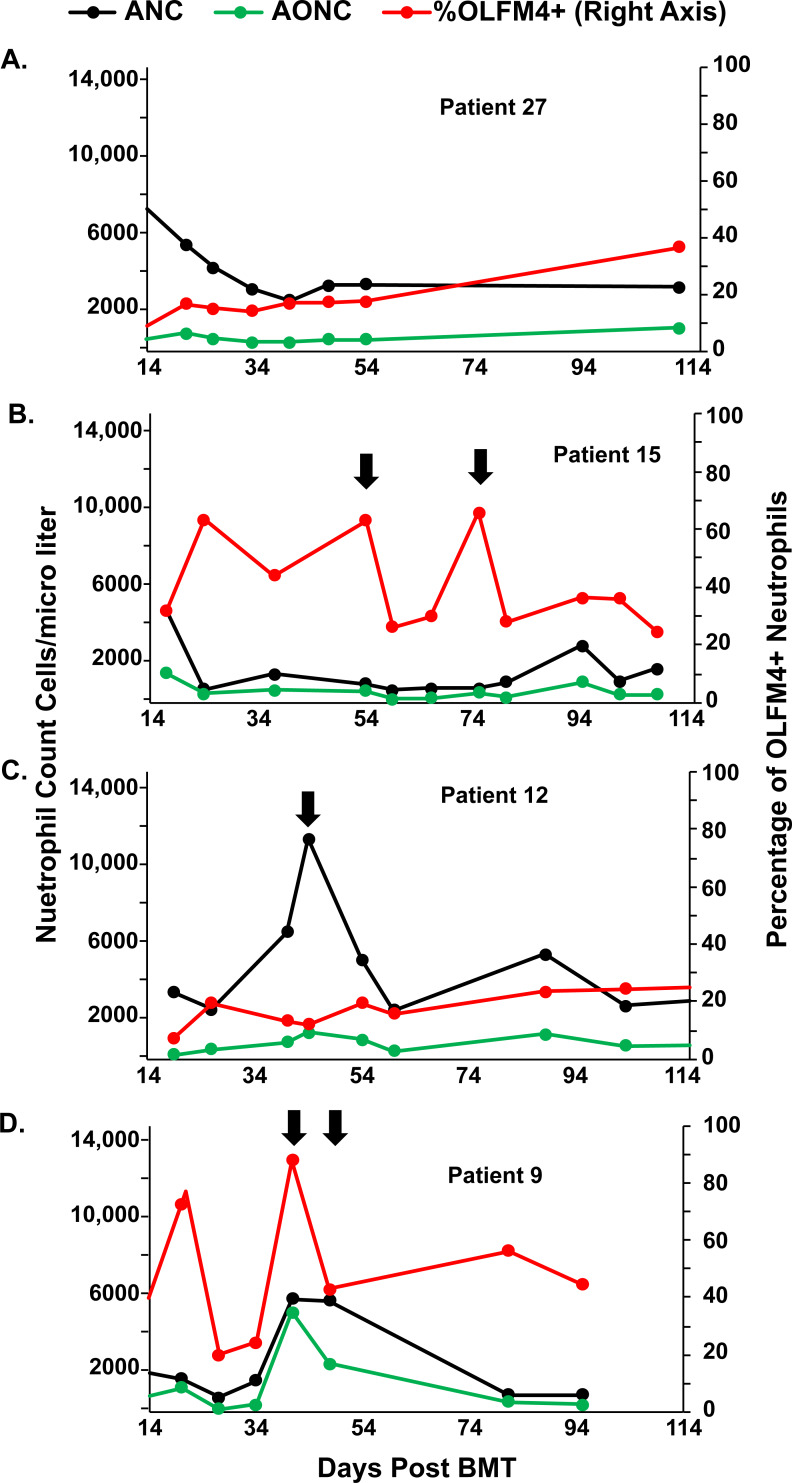
Variation in OLFM4 expression in patients following bone marrow transplantation. Four different patients were selected to show variation in percentage of OLFM4+ neutrophils. Arrows demark time points discussed further in the text. ANC = absolute neutrophils count (black), AONC = absolute OLFM4 neutrophil count (green) and %OLFM4+ = is the percentage of neutrophils that express OLFM4 (red). As demonstrated here, some patients had a stable ANC and AONC throughout their course (A). Others, showed an increase in only OLFM4+ neutrophils (B) or only OLFM4- neutrophils (C). Others, had a simultaneous increase in both (D).

There was wide variability in percentage of OLFM4+ neutrophils within individual patients in the weeks following bone marrow transplantation (Fig 2). Some patients had little change in the percentage of OLFM4+ neutrophils or the absolute OLFM4+ neutrophil count (AONC) throughout the BMT hospitalization (Fig 2A). Other patients varied the percentage of neutrophils that express OLFM4 but had little change in the AONC (Fig 2B) or kept the AONC essentially constant, even in the face of large spikes in the total neutrophil count (Fig 2C). Finally, we also noted that some patients decreased AONC while keeping absolute neutrophil count unchanged (Fig 2D). We tested for association between percentage of OLFM4+ neutrophils and total white blood cell count (R = -0.068, p = 0.14) and absolute neutrophil count (ANC) (R = -0.07, p = 0.11) and found no association.

**Fig 3:**
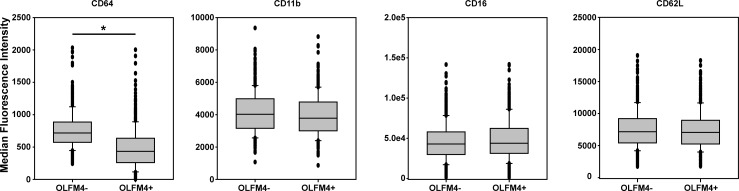
Neutrophil surface markers comparing OLFM4+ and OLFM4- neutrophils. Box and whisker plots showing median fluorescence intensity as well as 25^th^ and 75^th^ percental for each group of cells. *p<0.001.

We tested for differential expression of several cell surface markers on OLFM4+ and OLFM4- neutrophils (Fig 3). CD64 showed significantly less expression on OLFM4+ neutrophils, with a median MFI of 431 for OLFM4+ neutrophils and 761 for OLFM4- neutrophils (p<0.001). This finding was present at all time points post bone marrow transplant that we assessed. We assessed other surface markers as well, but found no difference between OLFM4+ and OLFM4- neutrophils for CD16 (median MFIs 42,984 and 43,955, p = 0.18), CD62L (medians MFIs 7,159 and 7,057, p = 0.41), or CD11b. Of note, CD11b trended toward different in the two groups with lower expression on OLFM4+ cells, but did not meet statistical significance (median MFIs 4,022 for OLFM4- and 3,779 for OLFM4+, p = 0.053).

**Fig 4:**
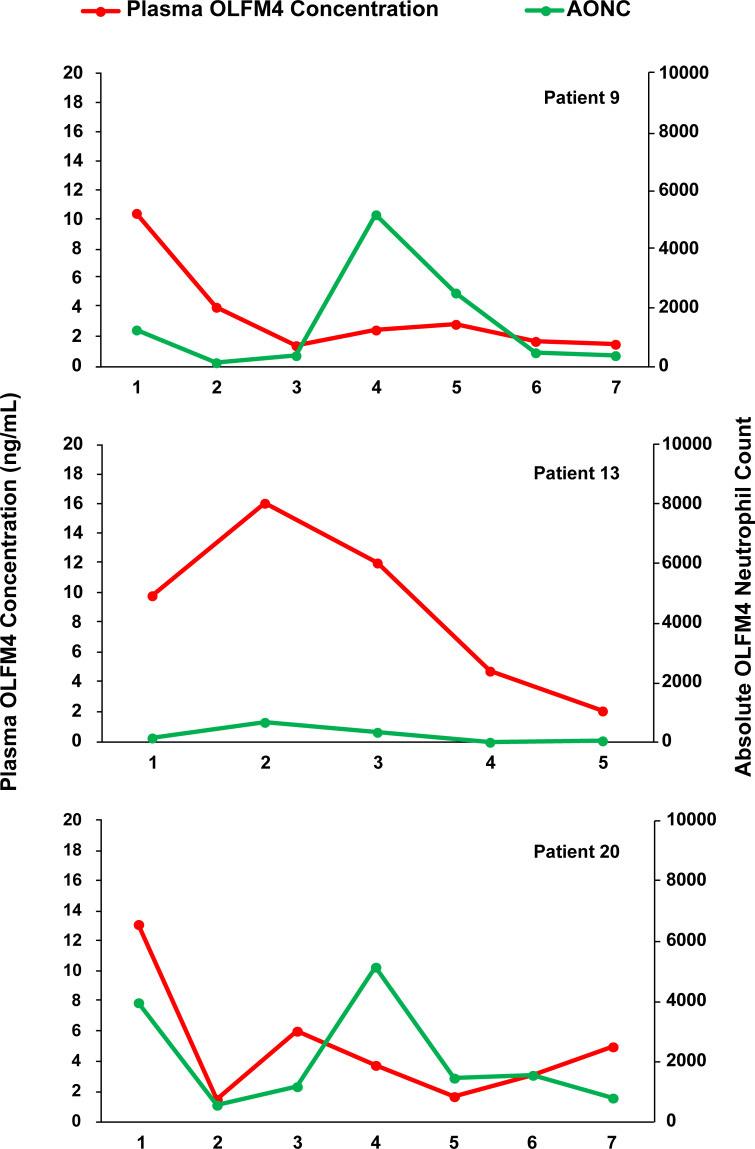
Plasma OLFM4 concentration relative to absolute number of OLFM4+ neutrophils. Three patients comparing number of OLFM+ neutrophils and plasma OLFM4 concentration.

We assessed plasma concentration of OLFM4 in relation to absolute number of OLFM4+ neutrophils to test if plasma OLFM4 came from neutrophils alone. We randomly selected 7 patients and measured plasma OLFM4 at weekly intervals and tested the association with absolute number of OLFM4+ neutrophils. Fig 4 shows representative data from 3 patients. We found no correlation between plasma OLFM4 and absolute OLFM4 positive neutrophils (R = 0.038, p = 0.80).

**Fig 5:**
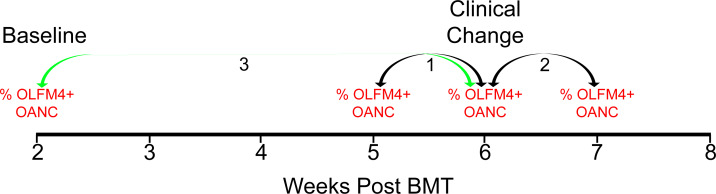
Three comparisons for change in OLFM4+ neutrophils relative to clinical change. Example of a patient who had a clinical change on week 6 post BMT. Arrows depict the three comparisons we made, 1-week prior to clinical change to week of clinical change. 2-Week of clinical change to week after clinical change. 3-Baseline level to week of clinical change.

Finally, we tested if changes in amount of OLFM4+ neutrophils were associated with clinically important outcomes. We chose to evaluate for complications that occur frequently following bone marrow transplantation including: sepsis (positive blood culture), acute kidney injury (AKI), hyperbilirubinemia, hypoxia (oxygen requirement), respiratory failure (requiring mechanical ventilation), acute GVHD and TMA. We evaluated for changes in both percentage of neutrophils that express OLFM4 and change in the total number of OLFM4+ neutrophils. There are no data available regarding what induces changes in OLFM4+ neutrophils and the amount of time it takes to change, so we made three different comparisons (Fig 5). First, the change in OLFM4+ neutrophils from the week before the change in clinical status to the week of the change in clinical status. Second, the change in OLFM4+ neutrophils from the week of the change in clinical status to the week following the change in clinical status. Third, change in OLFM4+ neutrophils from the first measurable level at 14 days post stem cell infusion, we refer to as baseline level, and the week of clinical change. We performed logistic regression to assess for associations between changes in the OLFM4+ neutrophils and these clinical events. Change in percentage of OLFM4+ neutrophils at any of the time points did not associate with any of the tested clinical outcomes (Table 2, upper). Fold change in absolute number of OLFM4+ neutrophils from baseline to week of clinical changes was associated with AKI and acute graft versus host disease, however, when changes in ANC were included in logistic regression, neither of these associations remained (Table 2, lower).

**Table 2:** Multiple regression analysis testing for association between percentage of neutrophils that express OLFM4 and clinical outcomes in BMT patients.

	Change in %OLFM4+ Neutrophils from week before to week of clinical change			Change in %OLFM4+ Neutrophils from week of clinical change to week after			Change in %OLFM4+ neutrophils from baseline to week of clinical change		
	**OR**	**95% CI**	**p value**	**OR**	**95% CI**	**p value**	**OR**	**95% CI**	**p value**
**AKI**	0.989	0.955–1.025	0.541	1.002	0.972–1.033	0.890	0.987	0.955–1.021	0.453
**Hyperbilirubinemia**^**1**^	0.996	0.996–0.963	0.815	0.990	0.960–1.021	0.531	0.994	0.958–1.031	0.737
**O2 requirement**	1.038	0.996–10.82	0.074	1.011	0.980–1.044	0.484	1.038	0.997–1.080	0.072
**Mechanical Ventilation**	1.016	0.971–1.064	0.488	1.040	0.996–1.086	0.076	1.027	0.982–1.075	0.244
**Positive Blood Culture**	0.999	0.949–1.051	0.960	1.018	0.967–1.071	0.504	1.017	0.964–1.073	0.536
**GVHD**	1.011	0.957–1.068	0.691	0.976	0.923–1.033	0.401	1.031	0.980–1.084	0.243
**TMA**	1.056	0.990–1.126	0.095	0.956	0.911–1.004	0.073	1.036	0.982–1.094	0.196
	Change in AONC from week before to week of clinical change			Change in AONC from week of clinical change to week after			Change in AONC from baseline to week of clinical change		
	**OR**	**95% CI**	**p value**	**OR**	**95% CI**	**p value**	**OR**	**95% CI**	**p value**
**AKI**	0.708	0.247–2.027	0.518	1.198	0.490–2.933	0.691	0.372	0.144–0.963	0.042*
**Hyperbilirubinemia**^**1**^	0.958	0.369–2.487	0.929	0.756	0.287–1.989	0.569	1.095	0.406–2.949	0.857
**O2 requirement**	1.470	0.462–4.680	0.512	0.926	0.345–2.487	0.878	2.502	0.868–7.215	0.089
**Mechanical Ventilation**	0.875	0.254–3.017	0.832	3.812	0.968–15.005	0.056	1.114	0.356–3.491	0.852
**Positive Blood Culture**	0.399	0.104–1.526	0.179	2.056	0.463–9.126	0.341	0.809	0.226–2.889	0.743
**GVHD**	2.290	0.415–12.632	0.340	0.912	0.152–5.467	0.919	5.433	1.609–18.345	0.007*
**TMA**	1.628	0.151–17.568	0.686	0.846	0.166–4.312	0.840	1.036	0.261–4.104	0.960

* These findings were no longer valid when ANC was included in regression analysis.

## Discussion

This study is the first large longitudinal study of OLFM4+ neutrophils in pediatric patients at high risk for clinical complications following BMT. Previous studies have evaluated only healthy individuals over a long period of time showing little change in OLFM4 expression[8]. Here, we have shown that the percentage of OLFM4+ neutrophils can change over time in an individual patient, in measurements just one week apart. This study was initiated because we previously showed patients admitted with septic shock and high percentage of OLFM4+ neutrophils had worse outcomes. These current findings show that patients can change the percentage of OLFM4+ neutrophils from week to week and suggest that those septic patients with high percentage of OLFM4+ neutrophils could have been induced to express OLFM4 in a greater percentage of cells, rather than maintaining genetically determined levels of expression.

We first determined if host factors were responsible for determining percentage of OLFM4+ neutrophils. To examine this, we evaluated the last sample before myeloablative therapy and the first post engraftment level. There was no correlation between pre and post BMT percentage of OLFM4+ neutrophils. From the first detectable neutrophils to 100 days post engraftment, there was no difference in mean percentage of OLFM4+ neutrophils on any given week when evaluating all samples together (Fig 1) suggesting there is not general trend toward increase or decrease expression following bone marrow engraftment. There was also no association with age and OLFM4 expression. Genetic background does seem to play a role in OLFM4 expression as average percentage of OLFM4+ neutrophils is lower in Caucasian patients. Future studies of OLFM4 expression will need to take genetic background into account.

The variation in percentage of OLFM4+ neutrophils is not associated with changes in the white blood cell compartment. We tested for association with total WBC count and ANC and neither correlated with percentage of OLFM4+ neutrophils. Interestingly, in some cases ANC and AONC seem to be regulated independently of each other. For example, in patient 12 (Fig 2C) on day 40 there was a dramatic spike in ANC, but little increase in AONC. In contrast, in patient 9 (Fig 2D) on day 40 there is an increase in ANC that can be almost completely accounted for by OLFM4+ neutrophils. In the same patient seven days later, only half of the neutrophils are OLFM4+. Thus, based on these preliminary data, there may be circumstances when ANC and AONC are independently regulated. This may suggest these neutrophil subpopulations may have different effector functions but requires further testing.

We also investigated if cell surface markers were different between OLFM4+ and OLFM4- neutrophils, which may imply these neutrophils differ in expression of other proteins and functions beyond OLFM4 expression. Previously, Welin and colleagues showed that there was no association between CD177 and OLFM4 expression [7]. These authors evaluated CD177 because it is the only cell surface marker that clearly differentiates significant subsets of CD177 positive and negative neutrophils. We evaluated for other important neutrophil cell surface markers. There was no differential expression of CD16, CD11b, or CD62L between OLFM4+ and OLFM4- neutrophils. CD64, however, was decreased in OLFM4+ neutrophils relative to OLFM4- neutrophils.

CD64, also known as Fc γ receptor I, is a transmembrane glycoprotein that is a high-affinity IgG receptor found on neutrophils and macrophages [16]. In neonatal patients, CD64 expression has been used as a marker for neutrophil activation and a sepsis marker [17]. Two markers for neutrophil activation (CD11b and CD62L), however, were not differentially expressed between OLFM4+ and OLFM4- neutrophils. Previous studies have compared OLFM4+ and OLFM4- neutrophils relative to phagocytosis, transmigration, and apoptosis and found no difference between the groups [7]. Thus, the functional significance of lower expression of CD64 on OLFM4+ neutrophils is unclear at this time. However, the finding of differential expression of surface markers suggests that these neutrophils differ by more than OLFM4 expression alone and may differ in expression of other genes as well.

In our previous study of septic patients, increased plasma OLFM4 level was associated with worse outcomes [13]. Expression of OLFM4 by stem cells within the crypts of the intestinal mucosa has also led to the evaluation of plasma OLFM4 for prognostic potential in gastrointestinal tumors [18, 19]. A recent study in mice demonstrated that injured cells within the renal tubular epithelial cells also produce OLFM4 [20]. To test neutrophil contribution to plasma OLFM4 levels we measured plasma OLFM4 concentration at the same time as AONC over several weeks in multiple patients and found no association. Given BMT patients frequently have inflamed gastrointestinal mucosa and kidney injury, the gut and kidney could also be a sources of OLFM4 in this patient population. Further studies of OLFM4 will be needed to isolate potential sources of plasma OLFM4 and the significance of fluctuations in plasma concentration.

In our previous study in patients with septic shock, a high percentage of OLFM4+ neutrophils was associated with greater organ injury and death [13]. We were, however, unable to identify if the increased OLFM4 was because those patients always expressed high levels of OLFM4, or if high expression was induced by infection and organ injury because we only collected samples at the time of admission when patients were already critically ill. In the current study, we sampled patients serially, in order to test for association between clinical change and changes in expression of OLFM4. As this is the first report showing changes in the percentage and number of OLFM4+ neutrophils, we were unsure what induced changes and how long it took for these changes to occur. To address this, we assessed for the change in clinical status with change in percentage of OLFM4+ neutrophils and the AONC. We then compared percentage of OLFM4+ neutrophils and AONC at three different time points described above (Fig 5). We did not detect an association between percentage of OLFM4+ neutrophils and infection (positive blood culture). It is notable that several associations between increased oxygen requirement and need for mechanical ventilation in patients with increased OLFM4 expression, as increased OLFM4 expression has previously been associated with lung injury in adult ARDS and pediatric RSV infection [9, 10]. However, given the small number of patients and the number of comparisons made, these findings will need to be test further in a study looking more specifically at lung injury and OLFM4 expression. We also noted two associations between increase in AONC when compared to baseline AONC for AKI and acute GVHD. However, when we included ANC into the logistic regression these findings were no longer valid as these associations likely arose from the low ANC at baseline compared to weeks later when the clinical change occurred.

Overall, we were surprised by the degree of variability in OLFM4 expression from week to week in patients following BMT, since previous reports showed little change in OLFM4 expression overtime. This is a novel and interesting finding, but also confounded many of our comparisons because we only sampled from 50 patients, and changes in clinical status were too rare to find specific trends with the high degree of background of variability. We still hypothesize that changes in OLFM4 neutrophils have clinical importance, but this issue requires further study in a larger cohort. We do not know if this degree of variability can be seen in other diseases or if it is specific to bone marrow transplant patients. Unfortunately, there are few pediatric patient populations who undergo weekly blood draws that would allow a similar comparison. Furthermore, we sampled patients at weekly intervals, making it difficult to know the kinetics of change in percentage of OLFM4+ neutrophils (hours vs days). Further studies may help elucidate if changes in percentage of OLFM4+ neutrophils are simply transcriptional activation of a previously silenced gene or if OLFM4 expression marks a unique subpopulation of neutrophils determined at the time of differentiation in the bone marrow.

In conclusions, this is the first study to follow OLFM4 percentage serially in non-healthy humans, specifically in pediatric patients undergoing BMT. We found a great deal more variability within individual patients than previously reported. We also found a correlation between OLFM4- neutrophils and increased CD64, which merits further investigation to test if this differential expression translates to functional differences between the populations. The clinical significance of changes in OLFM4+ neutrophil population will require future studies in a larger cohort, and in patients with different clinical pathologies.

## Supporting information

S1 Fig Validation of anti-OLFM4 antibody. To ensure the OLFM4+ subpopulation identified by our flow staining was indeed OLFM4, we stained the same patient’s sample with anti-human OLFM4 antibodies from four different sources. Shown is histograms from peripheral blood that has been gated based on doublet exclusion and CD66b+ to mark neutrophils. The Santa Cruz poly clonal and in-house poly clonal were permeabilized with saponin based buffer. Abcam and Sino Biological antibodies were permeabilized with methanol.
(DOCX)

S1 Data (XLSX)

## References

[1] Nauseef WM, Borregaard N. Neutrophils at work. Nat Immunol. 2014;15(7):602–11. 24940954 10.1038/ni.2921

[2] Kolaczkowska E, Kubes P. Neutrophil recruitment and function in health and inflammation. Nat Rev Immunol. 2013;13(3):159–75. 23435331 10.1038/nri3399

[3] Schlapbach LJ, Straney L, Alexander J, MacLaren G, Festa M, Schibler A, et al Mortality related to invasive infections, sepsis, and septic shock in critically ill children in Australia and New Zealand, 2002–13: a multicentre retrospective cohort study. Lancet Infect Dis. 2015;15(1):46–54. 25471555 10.1016/S1473-3099(14)71003-5

[4] Fitzgerald JC, Weiss SL, Kissoon N. 2016 Update for the Rogers' Textbook of Pediatric Intensive Care: Recognition and Initial Management of Shock. Pediatr Crit Care Med. 2016;17(11):1073–9. 27749512 10.1097/PCC.0000000000000942 PMC5389123

[5] Beyrau M, Bodkin JV, Nourshargh S. Neutrophil heterogeneity in health and disease: a revitalized avenue in inflammation and immunity. Open Biology. 2012;2. 10.1098/rsob.120134 PMC3513838 23226600

[6] Deniset JF, Kubes P. Neutrophil heterogeneity: Bona fide subsets or polarization states? J Leukoc Biol. 2018;103(5):829–38. 29462505 10.1002/JLB.3RI0917-361R

[7] Welin A, Amirbeagi F, Christenson K, Bjorkman L, Bjornsdottir H, Forsman H, et al The Human Neutrophil Subsets Defined by the Presence or Absence of OLFM4 Both Transmigrate into Tissue In Vivo and Give Rise to Distinct NETs In Vitro. Plos One. 2013;8(7). 10.1371/journal.pone.0069575 PMC3726694 23922742

[8] Clemmensen SN, Bohr CT, Rorvig S, Glenthoj A, Mora-Jensen H, Cramer EP, et al Olfactomedin 4 defines a subset of human neutrophils. Journal of Leukocyte Biology. 2012;91(3):495–500. 22187488 10.1189/jlb.0811417 PMC3289394

[9] Brand HK, Ahout IML, de Ridder D, van Diepen A, Li Y, Zaalberg M, et al Olfactomedin 4 Serves as a Marker for Disease Severity in Pediatric Respiratory Syncytial Virus (RSV) Infection. Plos One. 2015;10(7). 10.1371/journal.pone.0131927 PMC4498630 26162090

[10] Kangelaris KN, Prakash A, Liu KD, Aouizerat B, Woodruff PG, Erle DJ, et al Increased expression of neutrophil-related genes in patients with early sepsis-induced ARDS. American Journal of Physiology-Lung Cellular and Molecular Physiology. 2015;308(11):L1102–L13. 25795726 10.1152/ajplung.00380.2014 PMC4451399

[11] Wong HR, Shanley TP, Sakthivel B, Cvijanovich N, Lin R, Allen GL, et al Genome-level expression profiles in pediatric septic shock indicate a role for altered zinc homeostasis in poor outcome. Physiological Genomics. 2007;30(2):146–55. 17374846 10.1152/physiolgenomics.00024.2007 PMC2770262

[12] Wong HR, Cvijanovich NZ, Allen GL, Thomas NJ, Freishtat RJ, Anas N, et al Corticosteroids Are Associated with Repression of Adaptive Immunity Gene Programs in Pediatric Septic Shock. American Journal of Respiratory and Critical Care Medicine. 2014;189(8):940–6. 24650276 10.1164/rccm.201401-0171OC PMC4098101

[13] Alder MN, Opoka AM, Lahni P, Hildeman DA, Wong HR. Olfactomedin-4 Is a Candidate Marker for a Pathogenic Neutrophil Subset in Septic Shock. Crit Care Med. 2016. 10.1097/CCM.0000000000002102 PMC5512699 27635771

[14] KDIGO AKI Work Group: KDIGO clinical practice guideline for acute kidney injury. Kidney Int. 2012;2(1):1–138.

[15] Matics TJ, Sanchez-Pinto LN. Adaptation and Validation of a Pediatric Sequential Organ Failure Assessment Score and Evaluation of the Sepsis-3 Definitions in Critically Ill Children. JAMA Pediatr. 2017;171(10):e172352. 28783810 10.1001/jamapediatrics.2017.2352 PMC6583375

[16] Hoffmeyer F, Witte K, Schmidt RE. The high-affinity Fc gamma RI on PMN: regulation of expression and signal transduction. Immunology. 1997;92(4):544–52. 9497497 10.1046/j.1365-2567.1997.00381.x PMC1364161

[17] Rudensky B, Sirota G, Erlichman M, Yinnon AM, Schlesinger Y. Neutrophil CD64 expression as a diagnostic marker of bacterial infection in febrile children presenting to a hospital emergency department. Pediatr Emerg Care. 2008;24(11):745–8. 18955911 10.1097/PEC.0b013e31818c2679

[18] Clemmensen SN, Glenthoj AJ, Heeboll S, Nielsen HJ, Koch C, Borregaard N. Plasma levels of OLFM4 in normals and patients with gastrointestinal cancer. Journal of Cellular and Molecular Medicine. 2015;19(12):2865–73. 26416558 10.1111/jcmm.12679 PMC4687705

[19] Yu L, Wang L, Chen S. Olfactomedin 4, a novel marker for the differentiation and progression of gastrointestinal cancers. Neoplasma. 2011;58(1):9–13. 21067260 10.4149/neo_2011_01_9

[20] Stark JE, Opoka AM, Mallela J, Devarajan P, Ma Q, Levinsky NC, et al Juvenile OLFM4-null mice are protected from sepsis. Am J Physiol Renal Physiol. 2020;318(3):F809–F16. 32068457 10.1152/ajprenal.00443.2019 PMC7099509

