# Supplementary material for: Longitudinal characterization of olfactomedin-4 expressing neutrophils in pediatric patients undergoing bone marrow transplantation
Source: PLoS One. 2020 May 29;15(5):e0233738. doi: 10.1371/journal.pone.0233738 (PMC7259555; doi:10.1371/journal.pone.0233738)
Supplement: S1 Fig — To ensure the OLFM4+ subpopulation identified by our flow staining was indeed OLFM4, we stained the same patient’s sample with anti-human OLFM4 antibodies from four different sources. Shown is histograms from peripheral blood that has been gated based on doublet exclusion and CD66b+ to mark neutrophils. The Santa Cruz poly clonal and in-house poly clonal were permeabilized with saponin based buffer. Abcam and Sino Biological antibodies were permeabilized with methanol. (DOCX) [file pone.0233738.s001.docx]

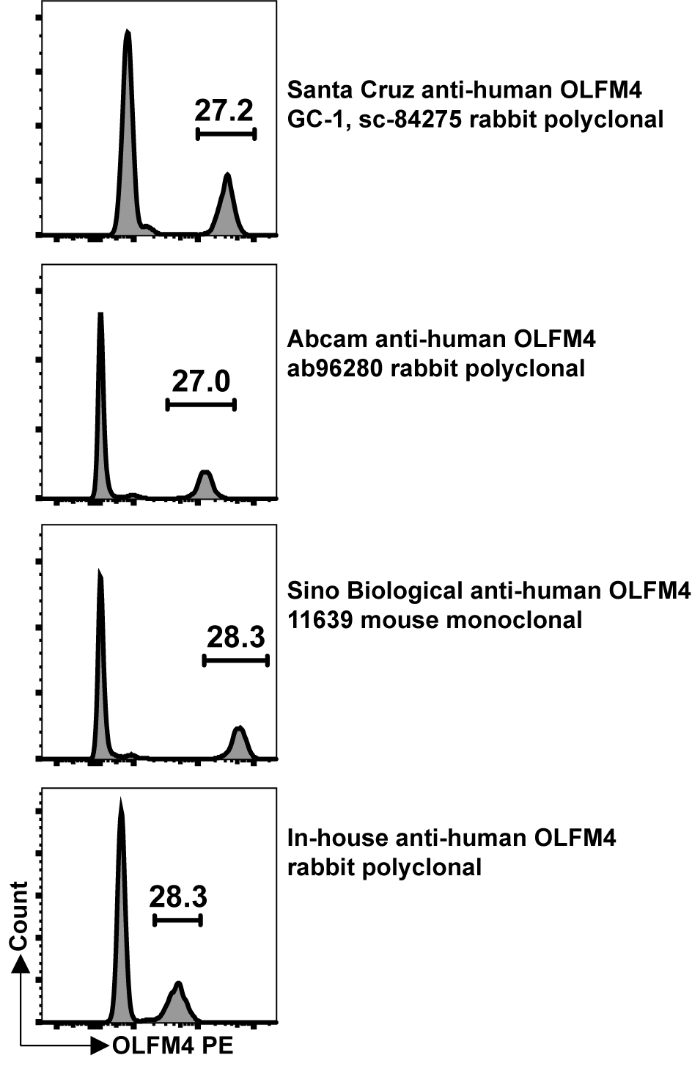


**Supplementary Figure 1. Validation of anti-OLFM4 antibody.** To ensure the OLFM4+ subpopulation identified by our flow staining was indeed OLFM4, we stained the same patient’s sample with anti-human OLFM4 antibodies from four different sources. Shown is histograms from peripheral blood that has been gated based on doublet exclusion and CD66b+ to mark neutrophils. The Santa Cruz poly clonal and in-house poly clonal were permeabilized with saponin based buffer. Abcam and Sino Biological antibodies were permeabilized with methanol.
